# Differential Neural Activation Patterns in Patients with Parkinson's Disease and Freezing of Gait in Response to Concurrent Cognitive and Motor Load

**DOI:** 10.1371/journal.pone.0052602

**Published:** 2013-01-30

**Authors:** James M. Shine, Elie Matar, Philip B. Ward, Samuel J. Bolitho, Mark Pearson, Sharon L. Naismith, Simon J. G. Lewis

**Affiliations:** 1 Parkinson's Disease Clinic, Brain and Mind Research Institute, The University of Sydney, Sydney, New South Wales, Australia; 2 School of Psychiatry, University of New South Wales, Sydney, New South Wales, Australia; 3 Schizophrenia Research Unit, South Western Sydney Local Health District, Liverpool, New South Wales, Australia; University of Toronto, Canada

## Abstract

Freezing of gait is a devastating symptom of Parkinson's disease (PD) that is exacerbated by the processing of cognitive information whilst walking. To date, no studies have explored the neural correlates associated with increases in cognitive load whilst performing a motor task in patients with freezing. In this experiment, 14 PD patients with and 15 PD patients without freezing of gait underwent 3T fMRI while performing a virtual reality gait task. Directions to walk and stop were presented on the viewing screen as either direct cues or as more cognitively indirect pre-learned cues. Both groups showed a consistent pattern of BOLD response within the Cognitive Control Network during performance of the paradigm. However, a between group comparison revealed that those PD patients with freezing of gait were less able to recruit the bilateral anterior insula, ventral striatum and the pre-supplementary motor area, as well as the left subthalamic nucleus when responding to indirect cognitive cues whilst maintaining a motor output. These results suggest that PD patients with freezing of gait are unable to properly recruit specific cortical and subcortical regions within the Cognitive Control Network during the performance of simultaneous motor and cognitive functions.

## Introduction

Freezing of gait (FOG) is a paroxysmal phenomenon that commonly affects patients in the advanced stages of Parkinson's disease (PD) leading to a high risk of falls and nursing home placement [1]. Despite its poorly understood pathophysiology [2], [3], widespread research has highlighted a number of common precipitating factors such as turning and initiating gait [2] as well as navigating narrow doorways [4]. Although perhaps not as frequent at triggering episodes many investigators have identified ‘dual-task performance' as a common trigger for FOG where patients freeze whilst having to walk and perform concurrent cognitive processing, [5]–[7]. Additionally, a number of studies have identified that patients with FOG have specific deficits on a variety of neuropsychological tests including attentional set-shifting and cognitive processing speed [8]–[10]. These findings raise the possibility that impaired cognitive processing might partially underlie those episodes of FOG related to dual-task performance, possibly mediated by disruption across frontostriatal networks [11].

One recent study has utilized functional magnetic resonance imaging (fMRI) to examine the neural correlates of dual-task performance comparing a group of PD patients with healthy controls [12]. In this study, patients were required to perform an over-learned finger-tapping task while concurrently performing a more cognitively demanding task, where they had to respond to the presentation of a specific letter on a computer screen. Both groups recruited the same specific network of brain regions in response to increased dual-task complexity, namely prefrontal and parietal cortices, widespread motor regions and the basal ganglia. These regions were also found to play an important role in another study exploring neural recruitment whilst performing the Wisconsin Card Sorting Task, a test known to probe set-shifting [13]. Although patients with PD and age-matched controls were able to recruit specific regions in the frontal cortex in response to task demand, they were unable to co-activate striatal regions. The authors concluded that impairments in nigrostriatal information processing may be responsible for the impairments in set-shifting specific to PD. However, these studies did not specifically explore differences between those patients with and without FOG.

To investigate this question, we utilized a virtual reality (VR) gait task with a variable amount of cognitive load in combination with fMRI. Using this approach we were able to examine the Blood Oxygenation-Level Dependent (BOLD) response whilst patients with and without FOG responded to cognitively demanding cues as they performed a motor task. Overall, we sought to determine whether an increase in cognitive load presented in the VR task was associated with a specific pattern of neural recruitment in cortical and subcortical regions and importantly, whether this response differed between those patients with and without FOG.

## Methods

### Patient details

The University of Sydney Human Research and Ethics Committee approved the study and written informed consent was obtained from each patient. All patients were screened for the study by scoring greater than 25 on the Mini Mental State Examination, and were thus considered to have the capacity to consent. In addition, the admission of patients to the study was also discussed with carers, where possible. The funders had no role in study design, data collection and analysis, decision to publish, or preparation of the manuscript.

Table 1 shows the demographic details of the patients who were all assessed in the clinically defined “off” state, having withdrawn from dopaminergic medications overnight. Although the entirety of the testing occurred in the “off” state, dopaminergic dose equivalence scores (in mg/day) were also calculated for each group, to ensure that subtle differences in regular medication state were not responsible for any group differences. Patients with FOG were screened for the study through the positive response to item three of the Freezing of Gait Questionnaire (*“Do you feel that your feet get glued to the floor while walking, making a turn or when trying to initiate walking (freezing)?”*) [14]. The response to this question has previously been shown to be a reliable screening tool for patients with FOG [15]. In addition, to confirm the presence of clinical FOG, patients were assessed in accordance with section III of the Movement Disorders Society Unified Parkinson's Disease Rating Scale (UPDRS III) [16] immediately prior to scanning. Specifically, patients were asked to perform a brief series of Timed Up-and-Go trials where they were required to make tight 180 degree turns to the left and right. Patients were deemed to suffer from FOG if they displayed one or more episodes of foot movement cessation during this brief assessment [17]. Patients that screened positively on the questionnaire but did not suffer from overt FOG in the clinical examination were not included in the analysis in either group.

**Table 1:** Demographic, neuropsychiatric and virtual reality characteristics.

	PD + FOG	PD − FOG	P value
*Demographics*			
Number	14	15	
Age	63.2+/−7.0	63.4+/−8.3	>0.1
Disease Duration (years) ^#^	5.9+/−3.6	4.9+/−2.9	>0.1
Hoehn and Yahr, stage	2.2+/−0.3	1.9+/−0.5	>0.05
UPDRS III ^#^ §	31.9+/−13.9	29.1+/−12.6	>0.05
FOG-Q total ^#^	10.5+/−3.2	1.7+/−2.2	<0.001
FOG-Q question 3 ^#^	2.8+/−0.8	0.0+/−0.0	<0.001
UPDRS item 46 ^#^	1.5+/−1.13	0.1+/−0.3	<0.001
*Neuropsychological Characteristics*			
MoCA ^#^	24.1+/−4.2	26.9+/−2.7	>0.05
HADS, total ^#^ §	11.5+/−4.6	4.6+/−2.3	<0.05
*Virtual Reality Paradigm*			
Modal footstep latency	0.6+/−0.4	0.7+/−0.2	>0.1
Out-of-sequence steps ^#^	27.2+/−35.4	9.4+/−9.1	<0.05
Delayed footstep responses ^#^	81.1+/−63.6	30.1+/−40.3	<0.05

UPDRS – Unified Parkinson's Disease Rating Scale; MoCA – Montreal Cognitive Assessment; MMSE – Mini Mental State Examination; HADS – Hospital Anxiety and Depression Scale; FOG-Q – Freezing of Gait Questionnaire. ^#^ – denotes t-test with unequal variance; § – denotes covariate entered into the 2nd level random effects design.

### Neuropsychological testing

Patients were assessed on the Montreal Cognitive Assessment (MoCA) [18] as a measure of general cognitive ability and the Hospital Anxiety and Depression Scale (HADS) [19] to measure affective symptoms.

### Virtual Reality Paradigm

A single 10-minute task was performed in the scanner, which consisted of a modified Stop-Signal task that was implemented in a VR environment [20], [21]. The virtual environment consisted of a straight corridor interspersed with environmental features, such as narrow doorways. The patients were positioned in the MR scanner so that they could clearly view the display on which the VR task was displayed, and their feet rested on a pair of MR-compatible footpedals [17], [21]. They were able to navigate a first-person view of the corridor by the use of the footpedals that were fixed to a board at the base of the MRI scanner. Forward progression within the VR environment was accomplished by the alternate depression of left and right footpedals, which required that the patient plantar flex the ankle of one foot ∼30 degrees below parallel, that activated a binary trigger mechanism. Navigation of the VR could only be achieved with alternating ‘physiological’ footstep sequences (i.e. left-right-left-right), which required the patient to dorsiflex the contralateral ankle to simulate a successful footstep. Forward progression did not occur during ‘out of sequence' steps (i.e. left–left or right–right), thus ensuring that movement through the VR environment was only associated with alternating left–right sequences. All footpedal responses were recorded for further analysis.

Patients were instructed to respond to ‘WALK’ cues by alternately tapping each footpedal in a rhythm consistent with their normal gait (∼2 Hz), and to cease this movement whenever a ‘STOP’ cue appeared. ‘WALK’ and ‘STOP’ cues were presented for 1.0 second in the bottom third of the screen at pseudorandom intervals in each block with a delay of at least 3.0 seconds prior to the presentation of a new cue. ‘WALK’ and ‘STOP’ cues were presented after a pre-set, yet variable, number of footsteps were taken, ensuring that patients were unable to predict the timing of the cue. If a patient stopped inappropriately (either intentionally or otherwise), the cue was re-presented on the screen after a delay of 3.0 seconds. Similarly, if a patient did not stop appropriately at a STOP cue, the cue was appeared every 3.0 seconds until the patient effectively stopped for a minimum of 1.5 seconds.

We manipulated task difficulty by introducing a second-order rule for stopping and walking based upon a modified version of the Stroop task. During the low cognitive load blocks, patients followed direct commands such as ‘WALK’ or ‘STOP’ (Direct WALK cue). In the high cognitive load blocks, WALK’ or ‘STOP’ cue were replaced with congruent (e.g. ‘BLUE’ written in blue) or incongruent (e.g. ‘BLUE’ written in green) Stroop color-words. Congruency of the Stroop color-words represented either a ‘WALK’ or a ‘STOP’ cue. For example, if congruent words represented ‘WALK’ then incongruent words represented ‘STOP’. Prior to scanning, participants were trained on the high and low cognitive load conditions until they demonstrated accurate responses to both Direct and Indirect ‘WALK’ and ‘STOP’ cues, which took less than four minutes in all subjects. The specific rule that a patient was expected to follow (Congruent or Incongruent Stoop words) was assigned to a patient randomly prior to the experimental training period.

The time-point (in seconds) associated with the presentation of a Direct or Indirect WALK cues were collected for further analysis. Based on each individual patient's VR cadence, a minimum of 30 and a maximum of 45 Direct or Indirect WALK cues were presented in the experiment. In addition, we also collected the subject-dependent time-points associated with the ongoing depression of each foot pedal. By using these time-points, we were able to calculate the specific between-footstep latency associated with the successful completion of two steps. The same process was also utilized to calculate between-footstep latencies for any out-of-sequence footsteps.

To ensure that the presence of motor arrests in the cohort of patients with FOG did not skew the analysis, we removed any cue associated with a long-latency footstep (defined as twice the modal footstep latency) [21] in the two footsteps following the presentation of either a Direct or Indirect cue. The modal footstep latency was taken to be a more robust measure of normal walking cadence than the average footstep latency [21], which has the potential to be skewed by the presence of prolonged footstep latencies associated with motor arrest [20]. As expected, the patients with FOG suffered from a higher number of these delayed footsteps immediately following cue presentation (t = 3.0, p<0.01). However no more than 9 of the 45 Indirect cues (4.7+/−2.3) were removed in any single patient. This measure ensured that only accurate responses to either form of WALK cue were analyzed.

The Direct WALK cues that followed a STOP cue (i.e. to recommence walking) were not included in the analysis, as these cues were felt to reflect a qualitatively distinct phenomenon (i.e. starting a motor sequence from stop, rather than processing an increase in cognitive load in the midst of a current motor task). Therefore, the difference between the responses to Indirect and Direct WALK cues represented a pure measure of cognitive demand required to process information whilst maintaining motor output.

### Neuroimaging analysis

#### Image acquisition

Imaging was conducted on a General Electric 3 Tesla MRI (General Electric, Milwaukee, USA). T2*-weighted echo planar functional images were acquired in sequential order with repetition time (TR)  = 3 s, echo time (TE)  = 32 ms, flip angle  = 90°, 32 axial slices covering the whole brain, field of view (FOV)  = 220 mm, inter-slice gap  = 0.4 mm, and raw voxel size  = 3.9 mm by 3.9 mm by 4 mm thick. High-resolution 3-D T1-weighted, anatomical images (voxel size 0.4×0.4×0.9 mm) were obtained for coregistration with functional data.

#### Image pre-processing

Statistical parametric mapping software (SPM8, Wellcome Trust Centre for Neuroimaging, London, UK, http://www.fil.ion.ucl.ac.uk/spm/software/) was used for image processing and analysis. Functional images were pre-processed according to a standard pipeline: a) scans were slice-time corrected to the median (17th) slice in each TR; b) scans were then realigned to create a mean realigned image and measures of 6 degrees of rigid head movements were calculated for later use in the correction of minor head movements; c) images were normalized to the Echo Planar Image (EPI) template; d) scans were then smoothed using an 8-mm full-width half-maximum isotropic Gaussian kernel; e) due to the increased risk of head movements in this clinical population, each trial was subsequently analyzed using ArtRepair [22] and trials with a large amount of global drift or scan-to-scan head movements greater than 1mm were corrected using interpolation. Trials with head-movements greater than 3mm or 3 degrees of movement were removed from the analysis. There were no significant differences in the average distance of scan-to-scan movement between the two groups (p>0.5). Following pre-processing, images were then imported into a number of 1^st^ level analyses.

#### 1^st^-level analysis

**Figure 1:**
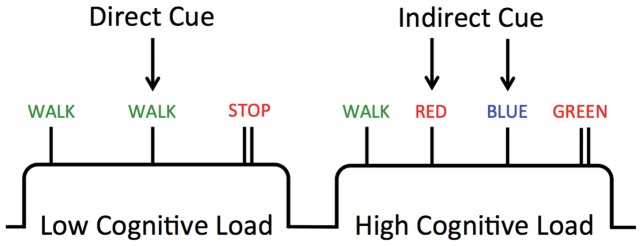
Experimental Paradigm. A graphical depiction of the experimental paradigm. Patients used a set of footpedals to navigate a virtual corridor while lying on their back in a 3T MRI scanner. During the navigation of the corridor, either Direct (e.g. the word ‘WALK’) or Indirect (e.g. the word ‘RED’ written in the colour *red*) cues were presented on the bottom 1/3 of a computer screen. Patients were asked to interpret these cues and determine whether to continue walking or to stop and await the next cue based on a pre-learned rule. The experiment was designed with interspersed blocks of low cognitive load (left block) and high cognitive load (right block), in which Indirect and Direct cues were presented, respectively.

Statistical parametric maps were calculated for each subject using a general linear model analysis within an event-related design in a fixed-effects analysis using SPM8 software. The high-pass filter was set to 128 seconds, stimulus durations for each cue were set to zero seconds and each experimental condition was convolved with the canonical haemodynamic response function in order to account for the temporal delay in the BOLD response. To create a contrast image that represented each patient's unique response to periods of increased cognitive load, the onset time (in seconds) of all Indirect WALK cues was then contrasted with the onset time (in seconds) of all Direct WALK cues (see Figure 1).

#### 2^nd^-level analysis

Individual contrast images from the first-level analyses were entered into a second-level random-effects design in order to determine the group-level effects of the condition of interest. We explored both the individual within-group patterns (using a one-sample t-test) and the between-group differences (using a two-sample t-test). As the level of self-reported affective symptoms differed significantly between the two groups, the HADS scores were entered into the model as a covariate so as to remove any substantial linear effects from the multivariate analysis. Whole brain voxel maps were subsequently corrected with a false detection rate of p<0.05 using XjView software.

#### Region of interest analysis

To further explore the directional patterns of the BOLD responses within the study, contrast images from the 1^st^-level analysis were analyzed using regions of interest (ROIs). Using the MarsBar toolbox in SPM8 [23], 8mm spherical ROIs were drawn around the peak voxels from the within-group and between-group second level T-maps (p<0.001 uncorrected; co-ordinates for these values are presented in Table 2). This analysis allowed us to explore whether any significant differences in the contrast value between the groups were due to a relative difference (i.e. both groups associated with a positive BOLD response, but one group with a relatively higher response) or difference in the overall response (i.e. one group with a positive BOLD response and the other with a negative response).

**Table 2:** Brain areas showing the largest increased BOLD response in the second level analyses comparing a Complex WALK cue with a Simple WALK cue.

Neural Region	x	y	z	Test statistic	Cluster size
R anterior insula	30	11	−17	−5.35	74
L superior frontal	−6	44	52	−4.83	34
L anterior insula	−33	20	−20	−4.50	32
L ventral striatum	−9	17	−5	−4.45	31
R ventral striatum	3	11	−8	−4.45	36
R pre-supplementary motor area	3	5	61	−4.30	35
L subthalamic nucleus	−12	−10	−5	−4.06	7

MNI co-ordinates for neural regions which displayed decreased BOLD response in the contrast between patients without FOG > patients with FOG when comparing a Complex WALK cue with a Simple WALK cue. T-statistics are presented for clusters corrected with a False Detection Rate of p<0.05.

Based on a number of *a priori* hypotheses of FOG [2], [3], we were also interested in the patterns of BOLD response within a number of key subcortical structures, including the head of the caudate nucleus (co-ordinates for the left and right ROI, respectively: -9 17 1 and 9 17 1), the putamen (-28 3 6 and 28 3 6), the ventral striatum (VS; -9 17 -5 and 3 11 -8), the globus pallidus internus (GPi; -16 -2 4 and 16 -2 4) and the subthalamic nucleus (STN; -11 -14 -3 and 11 -14 -3). The co-ordinates for the subcortical ROIs were defined *a priori* based on a study that traced basal ganglia ROIs using an EPI template similar to that used to normalize the functional scans in our study [24]. Care was taken to ensure that there was no overlap between the ROIs. The MarsBar toolbox was subsequently used to extract contrast values for each ROI dependent on the contrast of interest.

## Results

### Demographic and neuropsychiatric results

Demographic and neuropsychiatric data are included in Table 1. The two groups were matched for age (*t_27_* 1.17; *p*>0.1), dopaminergic dose equivalence (*t_27_* 0.98; *p*>0.1), disease duration (*t_27_* 1.14; *p*>0.1), Hoehn and Yahr (H&Y) stage (*t_27_* 1.98; *p*>0.05) and UPDRS III (motor) score (*t_27_* 0.54; *p*>0.1). In keeping with previous phenotypic descriptions, patients with FOG were more likely to self-report depression (*t_27_* 2.54; *p*<0.02) and anxiety (*t_27_* 2.79; *p*<0.01) symptoms on the HADS, however there was no discernible difference on the MoCA (*t_27_* 1.69; *p*>0.05), suggesting similar levels of general cognitive performance between the two groups.

Importantly, both patient groups walked with a similar modal footstep latency (*t_27_* 0.83; p>0.1), suggesting any group differences were not related to a more general motoric difference on performing the task. Patients with FOG did have more footsteps that were greater than twice the modal latency during the VR paradigm than patients without FOG (*t_27_* 2.42; *p*<0.02), and these patients also had a higher number of delayed footstep latencies in the two footsteps following a cognitive cue (*t_27_* 3.29; *p*<0.001). However, as stated above, the time-points related to these delayed footstep responses were removed from the dataset prior to the extraction of the time-points associated with the cognitive cues. Furthermore, no WALK cue presentation (Direct or Indirect) that occurred either two footsteps before or after a long-latency footstep was entered into the analysis. The removal of these cues ensured that significant between-group differences in the random-effects analysis were not due to a failure in processing the cognitive cue or related to motor arrest, such as a freezing event. Finally, the number of footsteps occurring at greater than twice the modal latency were not correlated with affective or neuropsychological performance (HADS score (*r* 0.135; *p*>0.1), the MoCA (*r* −0.03; *p*>0.1).

### Imaging results

#### Within-group similarities

**Figure 2:**
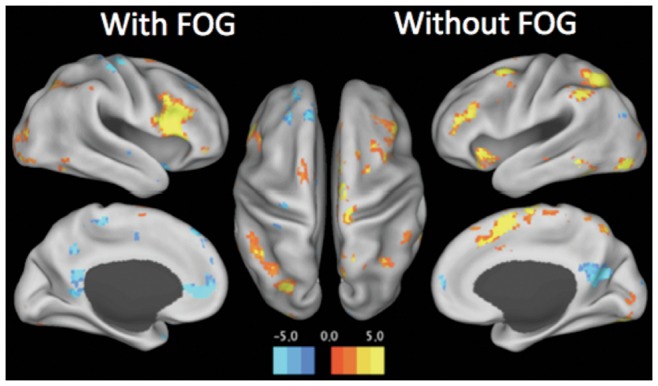
Within-group similarities. Cortical surface rendering of the major areas of increased and decreased BOLD response during the comparison of the Indirect WALK cue and the Direct WALK cue. A similar pattern was seen when comparing PD patients with and without FOG. Colour intensity on the graph represents the t-value obtained from the 2nd-level analysis of each group (corrected with a false detection rate of p<0.05).

Figure 2 shows the cortical BOLD response pattern associated with processing of an Indirect WALK cue in the VR paradigm. Patients with and without FOG showed increased relative BOLD signal in the bilateral dorsolateral prefrontal cortices, the bilateral posterior parietal cortices, the midline pre-supplementary motor area (pSMA) and the bilateral anterior insula (p<0.05 FDR). Significantly increased BOLD signal was also observed in the medial temporal lobes and the extra-striate visual cortex. Colour intensity on the graph represents the t-values extracted from the 2^nd^-level analysis for each group (p<0.05 FDR).

#### Between-group differences

**Figure 3:**
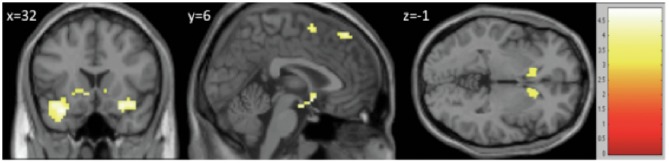
Differences between two groups (patients without FOG > patients with FOG). Slices of the brain representing the main regions of increased BOLD contrast in the comparison of patients without FOG > patients with FOG (corrected with a false detection rate of p<0.05) presented in the coronal (y = 6), sagittal (x = 32) and axial (z = −1) slices of a representative single T1 image. The major differences were found in the bilateral anterior insula, the bilateral ventral striatum and the pSMA. These differences were significant after controlling for group differences in motor severity, affective disturbance and impaired attentional set-shifting ability.

Figure 3 shows the regions of significantly decreased BOLD recruitment when comparing the 1^st^ level contrasts from the group of patients with FOG with the results from the group of patients without FOG (Indirect WALK cue > Direct WALK cue). Complete results from the 2^nd^ level analysis are presented in Table 2. After controlling for the severity of affective symptoms (HADS total score), patients with FOG had significantly less BOLD signal in the bilateral anterior insula (peak voxel from the right hemisphere: 30 11 -17 and *t* -5.35; left hemisphere: -30 14 -26 and *t* 4.48), the bilateral ventral striatum (left: -9 17 -5 and *t* 4.45 and right: 3 11 -8 and *t* 4.42), the left STN (-12 -10 -5) and the pSMA (peak voxel: 3 5 61, the cluster crossed the midline to include areas in both the left and the right hemisphere: x  = −4 to 7).

#### Region-of-interest analyses

**Figure 4:**
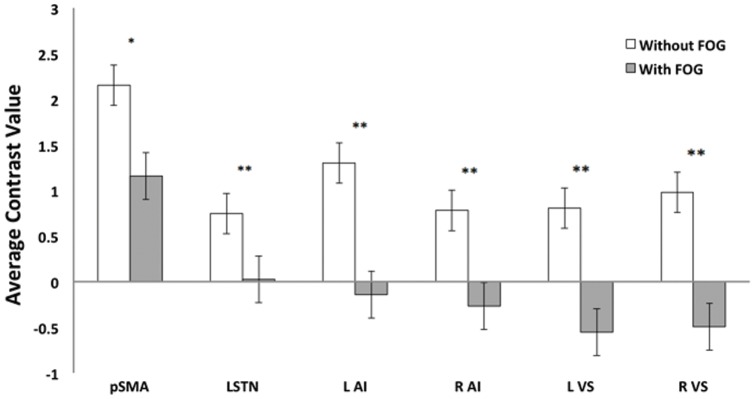
Results of the Region-of-Interest Analysis when viewing an Indirect > Direct cue (patients without FOG > patients with FOG). Results from the direct comparison of the contrast values from regions-of-interest between patients without FOG and patients with FOG. Error bars represent the estimated standard error for each value. Key: L  =  left; R  =  right; pSMA  =  presupplementary motor area; STN  =  subthalamic nucleus; AI  =  anterior insula; VS  =  ventral striatum. Significance levels: * – p<0.05; ** – p<0.01.

There were a number of ROIs that showed significant differences between the two groups of PD patients during the performance of a cognitively demanding cue (see Figure 4). While both groups showed a significant increase in BOLD response within the pSMA, the group without FOG had a significantly larger increase than the group with FOG (*t* 2.26, *p*<0.03). In contrast, patients with FOG showed a significant decrease in the BOLD response within the anterior insula, bilaterally (left: *t* 2.79, *p*<0.01; and right: *t* 2.92, *p*<0.01), the ventral striatum, bilaterally (left: *t* 3.38, *p*<0.001; and right: *t* 2.95, *p*<0.01) and the left STN (*t* 2.83, *p*<0.01), whereas non freezers showed an overall positive BOLD response in these subcortical nuclei.

## Discussion

This is the first study to show the specific neural correlates of the processing of increased cognitive load during a VR gait task in PD patients with and without FOG. The processing of an indirect cue while performing a VR gait task was associated with the recruitment of the dorsolateral prefrontal cortex, the posterior parietal cortices, the pSMA and extra-striate visual areas (see Figure 2). These regions are known to comprise a neural network, termed the Cognitive Control Network [25], which has been shown to co-activate in the presence of a variety of executive tasks [26] as well as during goal-directed behavior [27]. In a previous study, these same regions were found to be relatively over-activated in both PD patients and controls during dual-task performance [28], suggesting that the VR paradigm is able to robustly probe the networks responsible for the processing of cognitive tasks in PD.

Interestingly, the regions activated during periods of increased cognitive load have been previously implicated in a number of hypotheses developed to explain the pathophysiology of FOG [2], [3]. Indeed, a recent review of the neuroimaging literature in FOG concluded that FOG is likely to be due to a dysfunction in widespread frontal and parietal cortical regions [29], a finding that was consistent with the results of a single-subject fMRI study exploring FOG through the same VR paradigm utilized in this study [17]. In addition, these same regions have been found to show abnormal connectivity in a recent resting state analysis [30] and have also been associated with impaired grey matter integrity in volumetric studies [31], [32]. Taken together, these results suggest that there may be a deficit in the neural regions responsible for normal cognitive function in PD patients with FOG. In addition, the current study supports the notion that the VR gait paradigm may offer a valid technique for investigating the neural correlate of the freezing phenomenon further. Recent work employing an imagined gait paradigm has implicated the Mesencephalic Locomotor Region as being more active in patients with FOG [33]. Given that the VR gait paradigm requires an actual bipedal motor output, it is possible that future studies could be directed at exploring motor arrests to help confirm and extend such findings.

While a similar network of neural regions was recruited during periods of increased cognitive load in both cohorts of PD patients, comparison of the two groups showed that patients with FOG displayed relatively decreased BOLD responses in the bilateral anterior insula, the bilateral ventral striatum and the pSMA (see Figure 3 and Table 2), all of which are recognized as major nodes within the CCN [25]. Furthermore, these regions remained significantly different between the groups even after controlling for motor symptom severity and affective symptoms. This suggests that these differences in BOLD response are unlikely to be due to the effect of mood disturbance, motor impairment or region-specific synuclein load, but rather to an impairment in the mechanisms underlying dual-task performance caused by the same pathophysiological mechanism that is responsible for the presence of FOG.

The anterior insula and ventral striatum are important hubs within the mesolimbic frontostriatal loop [34], [35]. Both of these regions have been implicated in the assessment and execution of decision-making tasks [36], [37], as well as in the ongoing processing of feedback [38], [39]. Therefore, the specific patterns of relative hypo-activation in the BOLD signal may reflect a deficiency in rapid decision-making on the VR paradigm. The anterior insula and ventral striatum have also been implicated in the ‘switching’ of activity between different functional networks of the brain [40], [41]. This proposal is supported by resting state neuroimaging data, which shows that the anterior insula is correlated with slow temporal fluctuations in the cingulo-opercular network [42], [43], regions strongly associated with task switching and processing of environmental salience [41], [44].

The VR paradigm used here was specifically designed to probe rapid cognitive processing [20], [45] by requiring patients with FOG to continually decide whether to WALK or STOP whilst navigating an ecologically valid virtual environment. This rapid processing was also predicted to be more difficult during periods of dopaminergic deficiency, which has been shown to impair frontostriatal functional connectivity during a set-shifting task, albeit in healthy individuals [46]. As such, impairment in these regions provides preliminary evidence for the proposal that FOG arises from inability to properly switch activity between competing yet complementary neural networks [11]. If this interpretation is correct, then the BOLD response within the anterior insula would be expected to be increased during an episode of freezing, perhaps in response to increased environmental salience [41], [44].

The relative lack of BOLD response in the pSMA is consistent with a number of the previously proposed hypotheses attempting to explain FOG [2], [3]. The pSMA is involved in a multitude of behaviors, including voluntary movement, updating and sequencing of motor plans, response inhibition and task switching [47], [48]. Additionally, the pSMA is heavily interconnected with subcortical structures as part of the hyper-direct pathway of the basal ganglia [49], [50]. Recent research has shown that the pSMA provides inhibitory control over the firing of the STN, leading to a cessation of its facilitation of inhibitory output of the basal ganglia [51]. Interestingly, the output structures of the basal ganglia are associated with a relative decrease in BOLD response during optimal firing, due to their entry into low-energy synchronous oscillations [52]. As such, the relatively decreased BOLD response in the pSMA could reflect a loss of cortical inhibition over the subthalamic nucleus, “priming” the brain for a possible freezing episode.

While these results are intriguing, it is important to note that FOG is not triggered solely by cognitive events and is most commonly associated with turning or start hesitation [2], [3]. Freezing behavior often manifests after the navigation of environmental obstacles such as narrow doorways [4], or through the forced reduction of stride-length [53]. Other lines of evidence, such as the presence of concomitant panic attacks in patients with FOG [54], have also implicated dysfunction in limbic domains in the pathophysiology of the condition [55]. As this study did not attempt to examine these factors, any conclusions regarding the general pathophysiological mechanism of FOG must be constrained to those episodes directly related to the processing of cognitive load. While it is possible that dysfunction within a similar network is responsible for freezing behavior that is not related to cognitive load [11], further studies, such as the exploration of the cortical and subcortical structures associated with long-latency footsteps on the VR paradigm, are required to confirm this speculation.

## Conclusion

While episodes of FOG in PD are not always triggered by a transient increase in cognitive load, abnormalities in cognitive processing have been widely implicated in the pathophysiology of FOG in PD [2], [3], [28]. For the first time, we have shown that patients with FOG recruit a well-described neural network to deal with increases in cognitive load. Even after controlling for measures of motor disease severity and affective symptoms, patients with FOG showed reduced activation in a number of regions within the CCN, such as the anterior insula, ventral striatum, the pSMA and the STN. This pattern of impaired neural processing offers novel insights into the pathophysiological mechanism of FOG, which has been proposed to reflect an inability to effectively switch between competing, yet complementary neural networks under heavy task-demand [11]. This interpretation is strongly aligned with the results of a number of recent neuroimaging studies in patients with FOG [28], [30]–[32], which also implicate dysfunction within the frontoparietal regions of the CCN in the pathophysiology of FOG. Further research integrating results from multiple imaging modalities may help to further elucidate the underlying mechanisms of FOG in patients with PD.
